# Supplementary figures and images for: G Protein-Coupled Estrogen Receptor (GPER) and ERs Are Modulated in the Testis–Epididymal Complex in the Normal and Cryptorchid Dog
Source: Vet Sci. 2024 Jan 5;11(1):21. doi: 10.3390/vetsci11010021 (PMC10820011; doi:10.3390/vetsci11010021)

**Figure S1.** WB full membrane for Figure 3

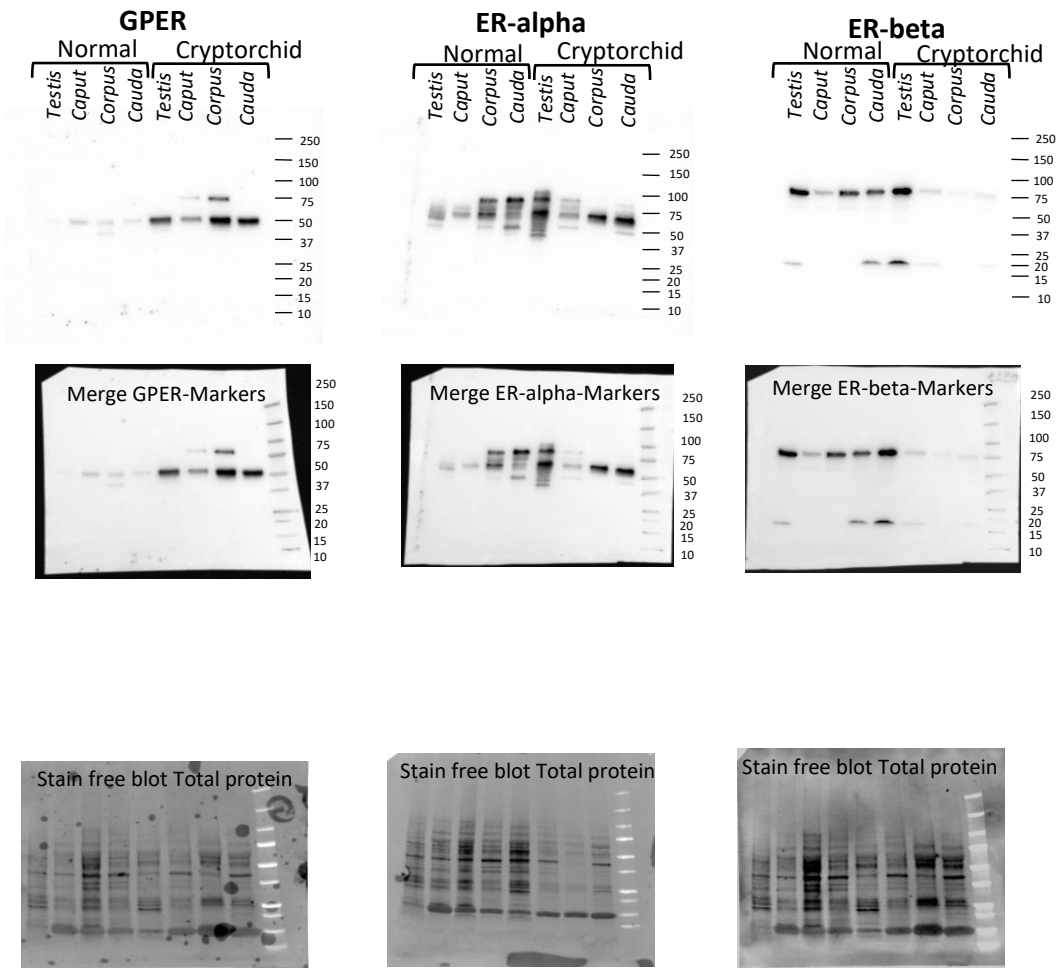

Figure S2. WB full membrane for Figure 4

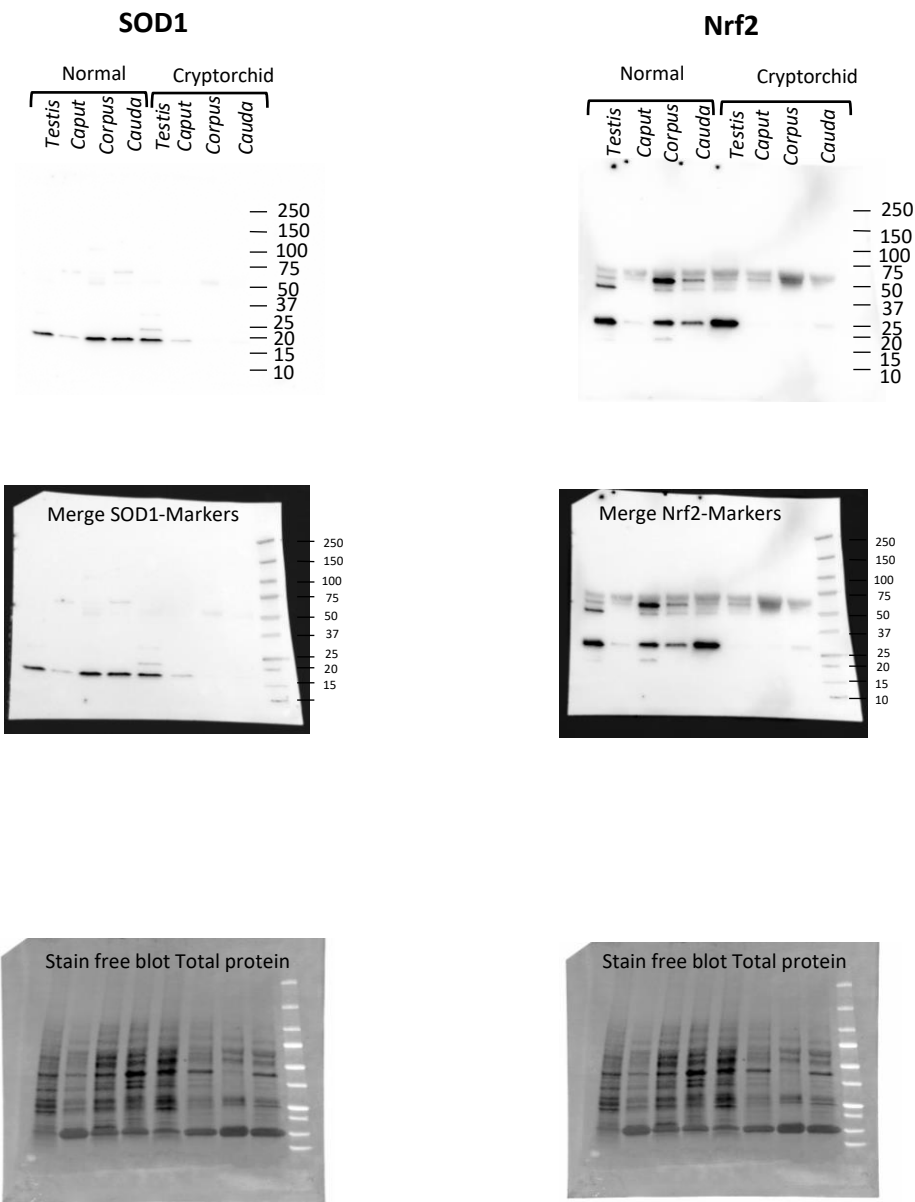

Supplement: Supplementary file 1 [file vetsci-11-00021-s001.zip › vetsci-2719300-supplementary.pdf]
